# Differential diagnoses of inguinal swellings: a case series of atypical diagnoses

**DOI:** 10.1093/jscr/rjae131

**Published:** 2024-03-13

**Authors:** Semen Ilgeldiev, David Tabidze, Soeren Stoeckel, Hagen Rudolph, Lutz Mirow

**Affiliations:** Department of General and Visceral Surgery, Klinikum Chemnitz gGmbH, 09116 Chemnitz, Germany; Department of General and Visceral Surgery, Klinikum Chemnitz gGmbH, 09116 Chemnitz, Germany; Department of General and Visceral Surgery, Klinikum Chemnitz gGmbH, 09116 Chemnitz, Germany; Department of General and Visceral Surgery, Klinikum Chemnitz gGmbH, 09116 Chemnitz, Germany; Department of General and Visceral Surgery, Klinikum Chemnitz gGmbH, 09116 Chemnitz, Germany

**Keywords:** groin swelling, inguinal swelling, inguinal hernia, groin pain

## Abstract

This case series highlights the importance of a thorough differential diagnosis in patients with groin swelling, often mistaken for inguinal hernias. It presents three patients with groin swelling initially suspected of having inguinal hernias but diagnosed differently upon further investigation. Patient 1 had a recurrence of endometrial adenocarcinoma in the right groin, Patient 2 had penile carcinoma with left inguinal metastasis, and Patient 3 had a metastasis of prostate carcinoma in the left groin. These cases underline the need to consider various pathologies beyond the common diagnosis of inguinal hernia. Accurate diagnosis requires a careful clinical examination and appropriate diagnostic tools, ensuring correct treatment.

## Introduction

The inguinal region is an anatomically complex area containing a multitude of structures. Swellings in this region are often interpreted as inguinal hernias, especially when they clinically present with typical symptoms of a hernia. Inguinal hernia is one of the most common diagnoses in patients presenting with groin swelling, leading to over 800 000 surgeries worldwide annually [1]. However, other pathologies can also produce similar clinical symptoms and should be considered in the differential diagnosis.

The literature reports various pathologies misdiagnosed as inguinal hernias. For example, Kojima *et al*. [2] highlight the importance of ultrasound examination in assessing patients with a groin mass, noting that such examination can help avoid unnecessary surgeries and identify latent inguinal hernias. Another report by Hwang *et al.* [3] identified a Nuck canal cyst as a rare anomaly of the female inguinal canal that can present similarly to a hernia.

In this case series, we present three patients who presented with symptoms of groin swelling. All were initially suspected of having inguinal hernias. However, further diagnostics led to three different diagnoses: an endometrioid adenocarcinoma, a penile carcinoma with inguinal metastasis, and a bone metastasis of a prostate carcinoma. These cases underscore the importance of thorough differential diagnosis and the use of appropriate diagnostic tools to make an accurate diagnosis and ensure appropriate treatment.

## Case report

We present a case series of three patients who presented at the Chemnitz Hospital with groin swelling and suspected inguinal hernias. A summary of the diagnostic and therapeutic timeline, as well as demographic characteristics, can be found in Table 1. The study was conducted in accordance with the Declaration of Helsinki and the ethical standards of the Chemnitz Hospital. The presentation and discussion of the cases, as well as the conclusions drawn, were guided by the CARE guidelines.

**Table 1:** Summary of the chronological sequence of diagnostics and therapy, as well as demographic characteristics.

	Case 1		Case 2		Case 3	
Age	70		73		67	
BMI	26,1		22		26,9	
Previous Illnesses	Moderately differentiated endometrioid adenocarcinoma of the endometrium, diagnosed 11 March 2020; atrial fibrillation, hypertension, epilepsy, history of appendectomy, history of tonsillectomy		Dialysis-dependent renal insufficiency, hypertension, vitamin D deficiency, shrunken kidneys, hyperuricemia, and bladder diverticula		Prostate carcinoma (first diagnosed in 2021), secondary malignant neoplasm of the bone, history of radiotherapy in 2021; history of appendectomy	
Medical History	Protrusion in the groin area, 10 × 15 cm, suspected inguinal hernia, differential diagnosis hematoma due to fall		Protrusion in the groin area, suspected inguinal hernia		Protrusion in the groin area, ~10 cm, suspected inguinal hernia	
Local Status	During the examination of the abdomen, a non-reducible mass of ~10 × 15 cm in the right groin area was diagnosed		During the physical examination, an irregular, indolent tumor in the left groin was noted		During the admission examinations, a non-movable mass of ~10 cm against the underlying tissue in the left groin area was diagnosed	
Course of Therapy/Diagnostics	Feb 22	Surgical consultation: ultrasound, CT abdomen	Mai 21	Inpatient surgical diagnostics: CT abdomen, urological consultation, ultrasound	Jun 23	Inpatient surgical diagnostics: excision biopsy
	Apr 22	Gynecological consultation			Jul 23	Inpatient surgical diagnostics: CT thorax/abdomen with contrast, excision biopsy
	Jul 22	Inpatient surgical diagnostics: CT abdomen, colonoscopy, puncture and core biopsy			Aug 23	Inpatient staging: FDG/PET-CT, MRI pelvis, MRI head, spine MRI
	Sep 22	Surgical consultation				
	Okt 22	Surgical consultation				
	Dez 22	Inpatient surgical treatment: incision of the cyst with drainage				
	Jan 23	Surgical consultation				
	Feb 23	Inpatient surgical treatment: puncture and drainage of the cyst				
	Mrz 23	Surgical consultation				
	Apr 23	Surgical consultation				
	Apr 23	Inpatient surgical treatment: Radical removal of the lymph cyst in the right groin area				

### Patient 1

The 70-year-old patient N presented in February 2022 at the surgical consultation. She presented with a protrusion in the right groin area, initially suspected to be an inguinal hernia. During abdominal examination, a 10 × 15 cm non-reducible mass was detected in the right groin area. An ultrasound examination revealed a fluid-filled mass. The patient had a history of a moderately differentiated endometrioid adenocarcinoma of the endometrium, for which she had undergone Wertheim surgery in March 2020.

**Figure 1:**
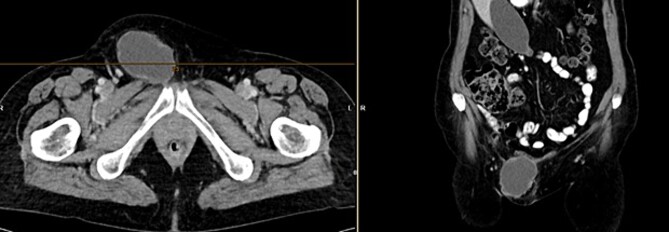
CT of the abdomen from 25 February 2022: oval encapsulated structure in the right groin area, most likely soft tissue hematoma, no active bleeding, DD enlarged lymph node.

**Figure 2:**
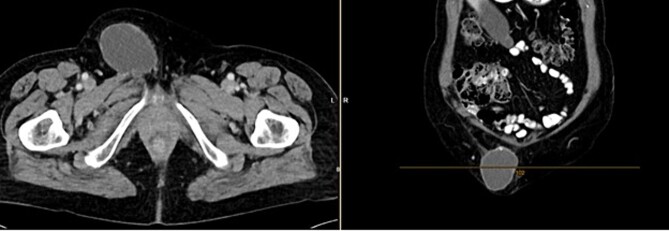
CT of the abdomen from 1 August 2022: size-progressive formation within the right inguinal hernia compared with the previous examination from 25 February 2022; no indication of tumor recurrence or metastasis-suspect lesions intra-abdominally, post-hysterectomy.

Further clarification was sought through a computed tomography (CT) scan of the entire abdomen (Fig. 1). The CT from 25 February 2022 showed an oval, encapsulated structure in the right groin area, most likely interpreted as a soft tissue hematoma. No active bleeding was observed. An enlarged lymph node was considered as a differential diagnosis. A follow-up CT on 1 August 2022 showed a size-progressive formation within the suspected right inguinal hernia compared with the previous examination in February (Fig. 2). There was no evidence of tumor recurrence or metastasis-suspect lesions intra-abdominally. A previous hysterectomy was also noted.

In September 2022, the case was discussed at the tumor board. There was no evidence of a tumor, and hernia surgery was recommended. The operation took place in December 2022. Intraoperatively, a cystic mass was identified. After incision of the cyst, a cytological examination was conducted. Cytology revealed a protein-rich aspirate with preserved squamous epithelial cells, including occasional atypical cells. Due to the low number of atypical cells, no further immunohistochemical examination was deemed necessary.

Upon recurrence of the cyst, another operation was performed, where the cyst was drained and cytologically examined again. Cytology showed a hematoma with signs of erythrocyte fragmentation and karyorrhexis of granulocytes. There was no evidence of malignancy in the examined material.

In April 2023, a cyst excision was performed. Histological examination revealed a fatty connective tissue portion with extensive infiltrates of a large adenocarcinoma in the right groin area. The carcinoma extended to the resection margin. Lymphangiosis carcinomatosa was also detected.

The final diagnosis was a recurrence of a moderately differentiated endometrioid adenocarcinoma of the endometrium in the right groin area.

### Patient 2

The 73-year-old patient M presented in May 2022 in our Central Emergency Department. He presented with a swelling in the left groin area that had been present for about 3 weeks. He reported normal bowel movements and micturition, and no B-symptoms were present. Physical examination in the emergency department revealed an irregular, indolent tumor in the left groin area. Based on these findings, a CT scan of the abdomen was ordered, and the patient was admitted for further diagnostics.

**Figure 3:**
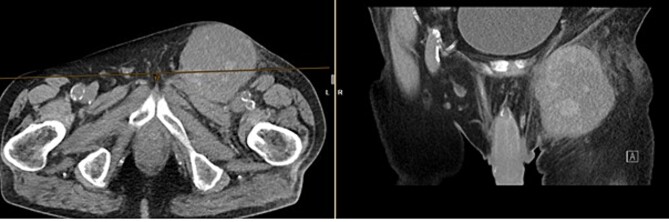
Finding from CT of the abdomen: highly suspicious malignant mass in the left groin area, with nearby satellite lesions, DD lymph nodes.

The CT of the abdomen showed a highly suspicious malignant mass in the left groin area (Fig 3). Additionally, nearby satellite lesions were detected, interpreted as lymph nodes. During a more thorough physical examination, a hazelnut-sized, highly suspicious malignant lesion was identified on the glans penis. Upon detailed anamnesis, the patient reported a previous circumcision surgery with carcinoma detection.

Based on these findings, the patient was referred to urology. The diagnosis was penile carcinoma with left inguinal metastasis.

### Patient 3

The 67-year-old patient P was admitted to our clinic in June 2023 due to a mass in the left groin area. The referral came from an outpatient urologist. The patient had a known history of prostate carcinoma from 2021, which had already metastasized and led to secondary malignant neoplasm of the bone. The patient had received radiotherapy in 2021 and had a history of appendectomy.

During the admission examination in June 2023, a ca. 10 cm non-movable mass was detected against the underlying tissue in the left groin area. An excision biopsy was conducted for histological clarification. Histological examination showed signs of a nodular, cell-rich scarring from the area of the left inguinal ligament. No evidence of malignancy, especially no evidence of carcinoma infiltrates, was found.

**Figure 4:**
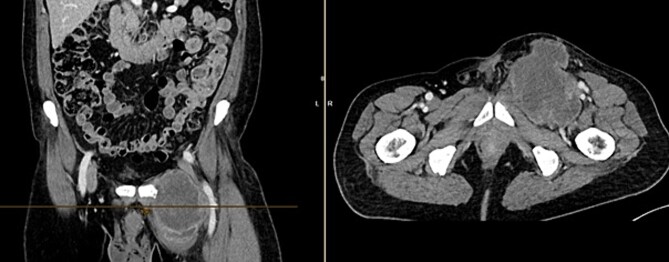
CT of the abdomen from 26 July 2023: size-progressive metastasis in the left groin with infiltration of the adductor muscles; no dynamics in the primarily osteoplastic diffuse osseous metastasis.

A month later, the patient presented again as the mass in the left groin area had increased in size. A CT (Fig 4) and another excision biopsy were performed. Histological examination revealed parts of a pleomorphic, most likely mesenchymally differentiated neoplasm. It was determined that the presence of a metastasis of the known prostate carcinoma could be ruled out. A myxofibrosarcoma was considered as a differential diagnosis, although the proliferation rate was rather low for this. Therefore, a reference pathology review was initiated.

The reference pathology confirmed the metastasis of an adenocarcinoma. The immunohistochemical profile of the tumor fit well in the clinical context with a metastasis of prostate carcinoma. Thus, the final diagnosis of a metastasis of the known prostate carcinoma in the left groin area was made.

## Discussion

The inguinal region is an anatomically complex area that houses a variety of structures. Swellings in this region are often interpreted as inguinal hernias. However, our case series emphatically demonstrates that not every swelling in the inguinal region is attributable to a hernia. The differential diagnosis of inguinal masses can be divided into five main groups: congenital anomalies, non-congenital hernias, vascular diseases, infectious or inflammatory processes, and neoplasms [4]. This spectrum of possible diagnoses underscores the importance of a thorough differential diagnosis.

Penile cancer is a rare malignancy occurring in Europe with an incidence of 0.1 to 0.9 per 100 000 male population [5]. In Germany, a total of 950 new cases were diagnosed in 2014 [6]. Most of these tumors are squamous cell carcinomas, with other cancer types such as malignant melanoma and basal cell carcinoma being less common [4]. Metastasis of penile cancer to other organs such as the bladder, prostate, and intestine has been described [2]. In patients with penile cancer, enlargement of the inguinal lymph nodes is often observed, which can be attributed to either metastases or infectious lymphadenitis. Disturbed lymphatic drainage from the lower extremities can lead to edema [7]. The diagnostic phase in cases of suspected penile cancer requires a thorough physical examination and cytological or histological confirmation of the diagnosis [5].

Prostate adenocarcinoma is the most common cancer in men, with about 50% of patients presenting with metastases at diagnosis [8]. Prostate adenocarcinomas metastasizing to inguinal lymph nodes without pelvic lymphadenopathy or other metastases are very unusual [9]. Therefore, prostate adenocarcinoma should be considered an important cause of an inguinal mass.

Endometriosis is a condition where benign tissue growth occurs outside the uterine cavity, similar to the endometrium [10]. The groin area is a rare site of endometriosis, with a frequency of 0.3–0.6% of all endometriosis patients [11]. The etiology of inguinal endometriosis is unclear, and it can be easily misdiagnosed as it often coexists with an inguinal hernia [12].

In summary, our case series highlights the importance of a thorough differential diagnosis in patients presenting with a swelling in the inguinal region. It is crucial to think beyond the most common diagnosis of an inguinal hernia and to consider other possible pathologies. This requires a careful clinical examination, supported by appropriate diagnostic tools, to establish an accurate diagnosis and ensure appropriate treatment.

## Conflict of interest statement

None declared.

## Funding

None declared.
